# Survey on the Environmental Risks of Bisphenol A and Its Relevant Regulations in Taiwan: An Environmental Endocrine-Disrupting Chemical of Increasing Concern

**DOI:** 10.3390/toxics11090722

**Published:** 2023-08-23

**Authors:** Wen-Tien Tsai

**Affiliations:** Graduate Institute of Bioresources, National Pingtung University of Science and Technology, Pingtung 912, Taiwan; wttsai@mail.npust.edu.tw; Tel.: +886-8-7703202

**Keywords:** bisphenol A, endocrine disruptor, environmental distribution, health risk, regulatory measure

## Abstract

Bisphenol A (BPA) has been identified as one of the endocrine disruptors or endocrine disrupting chemicals (EDCs). Due to its massive production (over 700,000 tons per year) and the extensive use of BPA-based plastics (i.e., polycarbonate and epoxy resin) in Taiwan, it was thus included as a toxic substance by the Ministry of Environment. This work surveyed the updated information about the production of BPA and its environmental distribution in Taiwan over the past decade. Furthermore, the regulatory strategies and countermeasures for managing the environmental risks of BPA by the Taiwan government were summarized to show the cross-ministerial efforts under the relevant acts, including the Toxic and Concerned Chemical Substances Control Act (TCCSCA), the Food Sanitation Management Act (FSMA) and the Commodity Inspection Act (CIA). The findings showed that most monitoring data were far below the acceptable risks. However, people may pose an adverse threat to the aquatic environment and human health via ecological and food chains. In addition, some countermeasures were further recommended to echo the international actions on environmental endocrine disruptors in recent years.

## 1. Introduction

**Figure 1:**
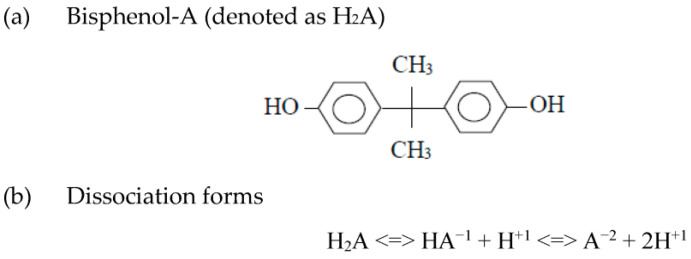
Molecular structure of (**a**) bisphenol A (denoted as H_2_A) and (**b**) its dissociation forms.

Bisphenol A (BPA), 2,2-bis(4-hydroxyphenyl) propane (CAS No.: 80-05-7), is an industrial chemical, which is mainly used in the manufacture of polycarbonate (PC) plastics and epoxy resins, and in many other applications, including polyvinyl chloride (PVC) antioxidant, plastic flame retardant and thermal papers [1]. In this regard, BPA is an important petrochemical substance with an annual global production of over 6.0 million tons in recent years [2]. As depicted in Figure 1, BPA is an organic compound with two phenolic groups, thus indicating its dissociation forms. Table 1 listed the main physicochemical properties of BPA [3,4], showing that it is a hydrophobic solid with high octanol-water partition coefficient (log Kow) value and low solubility in water. Therefore, the organic compound is favorable to its accumulation in sediments and tends to partition into water. In addition, the BPA-containing dust can create an explosive mixture with air under specific circumstances [5], thus posing a potential risk of exposure to airborne BPA in the workplace environment. It is noteworthy that BPA has been listed as one of the endocrine-disrupting chemicals (EDCs) due to its potential hormone-like and health effects on the reproductive system, immune system, neuroendocrine system, etc. [6,7]. Specifically, BPA has been shown to cause the possible transgenerational mode of action due to DNA methylation and epigenetic alterations during exposure in early life [8,9]. As reviewed by Sirasanagandla et al. [10], BPA could be associated with the modulation of autophagy, which may be important for medical treatment and drug discovery. Sirasanagandla et al. [11] also reviewed using natural products (i.e., plant extracts and natural compounds) for potential therapeutic treatment against adverse and harmful effects of BPA exposure.

Concerning the toxicity of BPA, there are several reviews on its health impacts [6,12,13,14,15,16]. On the other hand, BPA has been used to produce various daily life products, thus causing potential sources for human exposure. Therefore, the health risks associated with BPA exposure may occur mainly through food contamination from PC bottles and cans coated with epoxy resins [17,18,19] and thermal papers [20,21,22]. In addition, the critical parameters in the release of BPA from food and beverage containers included environmental, physical, and chemical factors like temperature, manufacturing process, food and packaging type, pH, mineral elements, repeated use, irradiation, washing, contact time, and using detergents [23]. Since the early 2000s, there have been considerable regulations in developed countries regarding the restrictions of BPA-derived products and food package materials, especially in infant breeding bottles and baby pacifiers [2,24]. In the European Union (EU), BPA has been included in Annex VI of the Classification, Labelling and Packaging Regulation in 2008. In 2018, BPA was included in the “Registration, Evaluation, Authorization, and Restriction of Chemicals” (REACH) Candidate List of Substances of Very High Concern (SVHC) by the European Chemicals Agency (ECHA). After that, BPA has been included as a toxic substance according to the related regulations in the EU, US, Canada, and Taiwan.

With the rapid demand for BPA-derived plastics, over 6250 thousand tons of raw BPA were produced in 2019 [2]. Furthermore, the global demand for BPA is expected to grow continuously at about 4% during the period of 2020–2030. The Asia Pacific region will have the largest BPA production, mainly due to the demand for PC and epoxy resin materials for manufacturing electrical/electronic products. Over the past two decades, Taiwan has been a key region in the production of BPA and BPA-derived products, accounting for over 10% of the total global BPA production capacity [2]. Therefore, the Taiwan government has begun to establish the cross-ministerial joint venture model to be more deeply involved with the preventive measures of EDCs and its survey on the environmental distributions and intake risks since the early 2000s. For example, the central competent authorities funded the projects for assessing dietary exposure to BPA [25,26,27,28].

The surveys on the environmental risks of BPA and its regulatory measures for mitigating human health in Taiwan were reviewed in previous studies [1,4]. It has been concluded that foodstuffs or diet was the major source of exposure to BPA. The foodstuff monitoring of BPA by the central competent authority (i.e., Food and Drug Administration, Ministry of Health and Welfare) in Taiwan has been regularly performed over the past decade. Based on the Taiwan National Food Consumption Database (https://tnfcds.nhri.edu.tw/, accessed on 18 August 2023), the intake showed below the tolerable daily intake (TDI) for BPA of 4 mg per kg of body weight per day, established by the European Food Safety Authority [16]. However, the information about the environmental distributions of BPA and its regulatory measures was progressive in the past decade. The present work will focus on an updated survey on the production of BPA and its environmental distribution in Taiwan. Furthermore, the regulatory strategies and countermeasures for managing the environmental risks of BPA by the Taiwan government were addressed to echo Taiwan’s sustainable development goals (SGDs) on environmental endocrine disruptors [29]. 

## 2. Data Mining Methods

In the present study, the main purposes of this survey were to summarize the updated information about the BPA production and consumption (i.e., production capacity, production amounts, exports and imports), the environmental distributions in the air, water and sediment media and regulatory countermeasures of the restricted use on the daily life products derived from BPA. In this regard, this survey work mined the open-accessed database from the official and relevant websites, which were further stated as follows:

- The updated information about the handling status of BPA

The updated data on the statistics of BPA handling (i.e., production capacity, production, export, and import) in Taiwan were mainly accessed on the websites [30,31,32], which were established by the central competent authorities and allied associations. They included the Toxic and Chemical Substances Administration (Ministry of Environment, Taipei, Taiwan), the Customs Administration (Ministry of Finance, Taipei, Taiwan), and the Petrochemical Industry Association of Taiwan.

- The updated information about environmental distributions of BPA

Using well-established websites like Google Scholar and Web of Science, an updated survey on the levels of BPA in the environmental media (i.e., air, water, and sediment) of Taiwan was reviewed. Herein, the environmental data from 2013–2022 were collected. It should be noted that these results were published by academic scholars based on the commissioned projects sponsored by the central competent authorities.

- Environmental policies and regulatory countermeasures for BPA management

To echo the international actions on environmental endocrine disruptors [33,34,35], the Taiwan government established a cross-ministerial platform for the environmental policies and regulatory countermeasures for EDCs management [36]. In addition, the relevant regulations for mitigating health risks from exposure to BPA, including the Food Safety and Sanitation Act, the Toxic and Concerned Chemical Substances Control Act, and the Commodity Inspection Act, were obtained from the official laws & regulations database [37].

## 3. Results and Discussion

### 3.1. A Survey on Bisphenol A (BPA) Production in Taiwan

#### 3.1.1. Overview of Production Processes for Bisphenol A (BPA) 

BPA (HOC_6_H_4_C(CH_3_)_2_C_6_H_4_OH) is commercially produced by acid-catalyzed condensation reaction of two moles of phenol (C_6_H_5_OH) and one mole of acetone (CH_3_COCH_3_) by the following stoichiometric equation.

2 Phenol + Acetone => BPA + H_2_O

**Figure 2:**
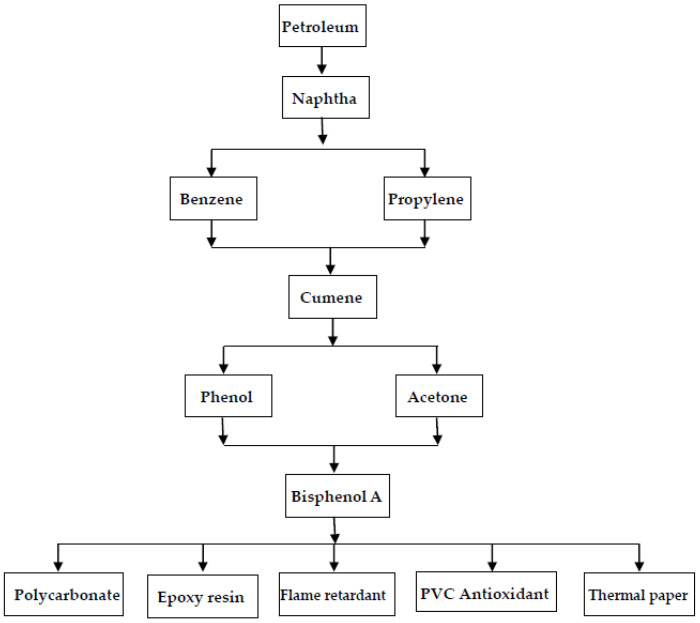
Flowsheet of BPA production process and its industrial applications.

These chemical feedstocks are produced from cumene (isopropylbenzene, C_6_H_5_CH(CH_3_)_2_), which is derived from the alkylation of benzene with propylene. In this regard, BPA is derived initially from petroleum refining for producing naphtha (a flammable hydrocarbon mixture or a fraction of crude oil), which is further converted into basic petroleum chemicals, including aromatic hydrocarbons (e.g., benzene) and olefins (e.g., propylene), by fractional distillation. Figure 2 shows the simplified flowsheet of the BPA production process and its industrial applications. The industrial applications or commercial uses of BPA will be further addressed in the following section. The traditional BPA production process has been based on a strong mineral acid catalyst, thus causing expensive corrosion-proof materials for construction and waste treatment. However, with the need for environmentally benign processes, cation exchange resin has now been widely used as an alternative catalyst [38], thus mitigating equipment corrosion and other waste/wastewater treatment problems. In commercial plants, the BPA product is typically isolated and purified from the reactor effluent using multiple crystallization processes. To optimize production efficiency, the solvents and unreacted acetone are further purified using distillation and recycled. Furthermore, an excess of phenol is used to achieve higher BPA selectivity.

#### 3.1.2. Overview of Commercial Uses of Bisphenol A (BPA)

BPA is mainly used as a raw material of polycarbonate (PC) and epoxy resin. Due to its high impact resistance and optical clarity, the former is traditionally achieved by a reaction with phosgene. It should be noted that a modern and environment-friendly PC production plant was operated by the Chi-Mei Company (Tainan, Taiwan) using a transesterification route [39]. Based on its excellent physical properties, PC can be made into a variety of common consumer goods, such as water bottles, sports equipment, safety glass (goggles), CDs (compact discs) and DVDs (digital versatile discs). By contrast, the latter is produced by a reaction with epichlorohydrin under basic conditions. It is used to line water pipes as coatings on the inside of many food and beverage cans, paints, coatings, adhesives, circuit packaging materials, and printed circuit boards (PCB). The other applications included the production of thermal paper coated with BPA (as a developing agent) and used in fax machines and sales receipts, tetrabromobisphenol A (a brominated flame retardant), and polyvinyl chloride (PVC) antioxidant in various products (including billboards signs, building materials, furniture, etc.). Therefore, the commonly used items or articles containing BPA, like plastic water bottles, baby bottles and food cans, may face leaching and contamination issues under abnormal environments like high temperature, acidic and/or basic conditions. The primary sources of exposure to BPA and their human health risks have been extensively reviewed in recent years [8,9,10,11,12,13,14,15,16], not discussed in this work.

#### 3.1.3. Status of Bisphenol A (BPA) Production in Taiwan

In Taiwan, there are three BPA production groups (i.e., Nan Ya Plastic Co., Taipei; Chang Chun Plastics Co., Taipei, and Taiwan Prosperity Chem. Co., Taipei) before 2021. Table 2 lists the BPA-manufacturing companies and their production capacities. In recent years, the actual BPA production was about 700,000 tons from the official database [31]. It should be noted that the Taiwan Prosperity Chem. Co. has been merged into the Chang Chun Group in August 2021.

On the other hand, the production capacities of BPA plants have been slightly increased by debottlenecking process in recent years. It means that Taiwan’s total BPA production capacity exceeded 840,000 tons per year. Table 3 summarized the statistics on the BPA production capacities in 2019 by the countries/regions [2,40]. It showed that Taiwan may be the No. 1 in the world based on the production capacity per capita, implying high risks when exposed to BPA in the environment. However, the actual production of BPA must be lower than its production capacity, depending on the international commodity market (or price) and domestic production demand for BPA and BPA-based plastics (e.g., PC and epoxy resin). Table 4 lists the amounts of imports and exports for BPA since 2010 in Taiwan [30]. As compared to the total BPA production capacity (seen in Table 2), about 30% of BPA production in Taiwan was exported to Asian countries like China, Thailand, and Japan.

### 3.2. A Survey on the Levels of BPA in Taiwan’s Environment

Potentially, any item/article that contained BPA or was made from BPA may emit more or less it to the environment, depending on several factors such as article category, service life and environmental stressors. As seen in Table 3, the Taiwanese people, especially those in the production plants of BPA and BPA-based products (including residents near these factories), may pose high risks of BPA exposure. Due to the large production and use of BPA in Taiwan, the government has funded academic scholars to monitor the BPA levels in the environment since the early 2000s. Under the cross-ministerial joint venture, the environment, health, agriculture, and industry authorities in the central government, including Ministry of Environment (MOE) [41], Ministry of Health and Welfare (MOHW) [28], Ministry of Economic Affairs (MOEA) [42], Council of Agriculture (COA) [43] and National Science and Technology Council (NSTC) [44,45], shall work together to develop preventive strategies to solve EDC issues like environmental distribution monitoring. In the previous study [1], the levels of BPA in the water and sediment environments before 2012 were summarized, indicating higher levels of BPA in river water and sediment samples from heavy industrial parks (e.g., petrochemical zone) and urbanized areas. In the following sections, an updated survey on the levels of BPA in Taiwan was reviewed according to the environmental media (i.e., air, water, and sediment).

#### 3.2.1. Ambient Atmosphere

According to the physicochemical properties of BPA, the high potential for its presence could be occurred in the particulate matter (PM) of the urban atmosphere. However, few studies on the levels of PM-bound BPA in the atmospheric environment were reported in the literature [46,47,48,49]. Although there were no reports of the BPA levels in the ambient atmosphere of the Taiwan area, two studies were to determine the ambient BPA levels for workers in the BPA-containing plastic manufacturing industry and to evaluate the workers’ health risk [50,51]. As studied by Chen et al. [50], the ambient BPA levels for workers in the BPA production plant (A) and PVC film manufacturing plants (B, C and D) were determined in the workplace environment. The findings showed that the workers in plant A exposed to high BPA levels ranging from 0.01 to 652.02 μg/m^3^, while the BPA levels in other plants B/C/D were very low values in the range of 0.00 to 1.78 μg/m^3^. In another research by Chao et al. [51], the BPA concentrations of the plant from inhalable dusts using optical grade PC material ranged from 32.28 to 44.97 μg/m^3^, which were significantly higher than those (16.16 to 19.39 μg/m^3^) of the plant using food grade PC material. Although the results of BPA concentrations in the airborne environment are lower than the maximum workplace concentration (MAK) value (5 mg/m^3^, measured as the inhalable dust fraction) in Germany, effective prevention measures and occupational exposure assessments should be established to protect the workers’ health and also reduce the BPA discharge into the outdoor environment [52].

#### 3.2.2. Water Environment

In the river system, the continuous water inflow from its tributaries may have crucial impacts on the water quality of the mainstream due to the pollution by run-off or discharge of fertilizers, pesticides, and synthetic chemicals at midstream and upstream. As reviewed by the previous studies [1,4], the concentrations of BPA in Taiwan’s river water bodies seemed to be higher than those in other countries or areas. For example, the monitoring results showed that about 60% of water samples were from the Kao-Pin River (the second-longest river in Taiwan) [53]. To improve the water quality of the Kao-Pin River, the Water Resources Bureau of the Kaohsiung City Government continually raised the rate of household connection to sewer systems, which included nine sewage treatment plants along this river. Concerning the environmental distribution of BPA, Chen et al. performed the concentrations of phenols (including BPA) in water sources (raw water). They treated water from 11 water treatment plants [54], showing that all data were below 60 ng/L. Chen et al. analyzed the concentrations of BPA in the water samples from the Tamsui River (northern Taiwan) [55], indicating 508 ± 634 ng/L (geometric mean = 303 ng/L) for 66 samples. Hsieh et al. investigated the concentrations of emergent contaminants (including BPA) in the Wuluo constructed wetland (southern Taiwan) [56], exhibiting that a maximal concentration of BPA was obtained to be 1733 ng/L. Chen and Chou measured the environmental concentration of BPA in the aquatic environment (i.e., river water and suspended solid samples) in southern Taiwan [44], finding that the concentrations of BPA in the surface water and suspended solid samples were 0.09–392 μg/L and 0.08–58 μg/L, respectively. Cheng et al. assessed the occurrence of BPA in tap water supplied through polyvinyl chloride (PVC), stainless steel, and galvanized pipes [57], verifying that BPA was not detected in most household water samples. Chou et al. explored the potential contributors to endocrine-disrupting activities in Taiwan’s surface waters from six river water systems [58], having a BPA concentration range of <0.01–725 μg/L. Gao et al. determined the concentrations of EDC (including BPA) in the drinking water treatment plants for evaluating their health risks [59], denoting that all data were lower than the method detection limit (MDL) value (i.e., 0.74 μg/L) both in raw and drinking water samples. Liu et al. assessed a variety of EDCs (including BPA) with estrogenic activity from the waters of the Wuluo River in southern Taiwan [45], containing BPA at 1384.6 ng/L (the highest concentration) in summer and 682.57 ng/L in spring. Dai et al. investigated the occurrence and treatment of EDCs (e.g., BPA) in Taiwanese drinking water sources (a total of 49 samples from 15 water treatment plants) [42]. The findings showed that most BPA concentrations were lower than 20 ng/L, but a maximum BPA concentration of 150 ng/L was measured. Based on the mentioned results [42,45,54,55,56,57,58,59], the high BPA concentrations in Taiwanese rivers, especially in the rivers by industrial wastewater discharge, may pose an adverse threat to the aquatic environment and human health via the ecological chain.

#### 3.2.3. Sediment

Along with the investigations on the environmental distributions of BPA in the aquatic environment, the sediments were often sampled and measured their levels. As studied by Chen et al. [55], the concentrations of BPA in the sediment samples from the Tamsui River (northern Taiwan) were determined to be 62.7 ± 92.2 ng/g w.w. (geometric mean = 26.0 ng/g w.w.) for 66 samples. Lee et al. performed the spatial-temporal distributions of EDCs (including BPA) in sediments from the Tamsui River system (northern Taiwan) to evaluate their risks to aquatic ecosystems and human health [41], showing that the concentrations of BPA in sediments ranged from 1 to 144 mg/kg-dw. The findings of the high BPA levels in river water and sediments were interactively observed in the sampling sites near industrialized and urbanized areas.

### 3.3. Regulatory Countermeasures for Managing the Environmental Risks of BPA in Taiwan

As mentioned above, BPA has been listed as one of the EDCs because it has weak estrogen-like activity, posing possible hazards to human health and environmental risks and listing it as one of the toxic chemicals or concerned substances. Therefore, various existing and additional regulations have been announced to control human health risks and environmental exposure to BPA in many developed countries [2]. Based on the integrated multimedia solutions for the toxic substance, the central governing authorities in Taiwan jointly promulgated the regulatory strategies for managing the environmental risks of BPA under various regulations in recent years. On the other hand, the potential alternatives to BPA in various applications were recently introduced to the industry [60,61,62]. For example, bisphenol S (BPS) has been commercially used as an alternative for BPA in thermal paper [61]. The following sections will summarize and discuss these preventive measures with relevance to the issues of exposing to BPA.

#### 3.3.1. Ministry of Environment (MOE)

BPA was listed as one of the toxic chemical substances under the Toxic and Concerned Chemical Substances Control Act (TCCSCA) on 31 July 2009. The central competent authority relevant to the TCCSCA refers to the Ministry of Environment. This toxic substance has been categorized into Class 4, which refers to those that have endocrine disruptor properties or environmental pollutants/chemicals which endanger human health. According to Article 8 of the TCCSCA, the toxicity and relevant information of Class 4 toxic chemical substances shall be reported to the local competent authority upon permission before the handling, which refers to such activities as the manufacture, import, transportation, use, storage or discarding of the toxic chemical substance. Furthermore, the handlers of toxic chemical substances shall make the reports and regularly report the records concerning the handling amounts of toxic chemical substances and their release quantities in accordance with Article 11 in the TCCSCA. Table 5 lists the production amounts and release quantities of BPA during 2017–2021 [31]. BPA may be present in the environment due to various emission sources from industrial manufacturing activities. In addition, the Ministry of Environment also announced the control concentration standard for BPA as 30 wt% based on the authorization of Article 11 in the TCCSCA. It means that the BPA-based substance will be recognized as a toxic chemical substance if containing more than 30 wt% BPA.

#### 3.3.2. Ministry of Health and Welfare (MOHW)

Because of the adverse effects of BPA on human health through the food chain, the central competent authority in Taiwan (i.e., (MOHW) stipulated the maximal limits of total BPA (0.6 ppm) from various food containers/utensils (except infant breeding bottles) with making them by PC plastic in the regulation (“Sanitation Standard for Food Utensils, Containers and Packages”) under the authorization of the Food Sanitation Management Act (FSMA) in 2013. According to Article 5 of the regulation, infant feeding bottles made of plastics shall not contain BPA, meaning the ban on PC use. It should be noted that these requirements were in accordance with international trends [16]. Although many reviews or surveys have found trace amounts of BPA in the food media [15,16], the current dietary exposure in Taiwan for the general population should be far below the acceptable risks [25]. For example, the concentration levels in the various food samples were trace amounts of BPA (ranging from the detection limits to parts of a billion) based on the studies by Chang et al. [28] and Lee et al. [63].

#### 3.3.3. Ministry of Economic Affairs

The central competent authority for governing the affairs of industry and trade in Taiwan is the Ministry of Economic Affairs (MOEA). Regarding the management of BPA-containing articles and commodities, the implementation agency under the MOEA is the Bureau of Standards, Metrology & Inspection (BSMI). In response to the national plan for implementing environmental hormone management since 2010, the survey measures were regularly performed by the central competent bureau. Under the authorizations of the Commodity Inspection Act, the BSMI has issued the National Standards of the Republic of China (CNS) for commodities (e.g., toys) to ensure consumer rights. The limits of BPA had been set to be 0.04 mg/L and 50 ppm (μg/g) for the toys (CNS 4797) in 2020 and thermal paper (CNS 15477) in 2013, respectively.

## 4. Conclusions and Recommendations

Bisphenol A (BPA) has been widely used in the massive production of plastic products and their additives but is probably one of the most prevalent synthetic endocrine-disrupting chemicals (EDCs) or xenoestrogens. Therefore, the environmental and health risks from exposing to BPA have become an important issue around the world. This issue is of particular concern to Taiwan because this country/region could be the No. 1 based on the production capacity per capita. Furthermore, the regulatory strategies and measures for managing the environmental risks of BPA by the Taiwan government were addressed to echo the international actions on environmental endocrine disruptors. Although most of the monitoring data showed far below the acceptable risks, the high BPA concentrations in Taiwanese rivers, especially in those by industrial wastewater discharge, may pose an adverse threat to the aquatic environment and human health via the ecological chain.

Although the Taiwan government has adopted several countermeasures through cross-ministerial efforts over the past decade, some recommendations for reducing the health risks of exposing to BPA were further addressed as follows:

- Expanding the regulatory restrictions on using BPA-derived commodities, especially for baby/children products like toys.
- Establishing the monitoring systems for BPA and other EDCs in the atmospheric environment nearby the industrial parks, especially for the production plants of BPA and its derived products like PC and epoxy resin.
- Examining the health risk assessments or occupational exposure assessments for the workers in the production plants of BPA, PC and epoxy resin.
- Promoting BPA-free products, including thermal/carbonless receipts, water bottles and food cans.
- Analyzing the economic or social impacts of the BPA ban in Taiwan, especially for the petrochemical industry in the productions of BPA, PC and epoxy resin.
- Further research is needed to understand better the environmental fate and transport of BPA in Taiwan. This includes studies on the sources and pathways of BPA contamination in the environment and the potential risks to aquatic organisms and ecosystems.
- More research is needed to better understand the potential health effects of BPA exposure, particularly in vulnerable populations such as pregnant women and children. This includes studies on the mechanisms of BPA toxicity and the development of biomarkers for BPA exposure.

## Figures and Tables

**Table 1:** Main physicochemical properties of bisphenol A (BPA).

Environmental Property	Unit	Value	Comment
Molecular weight	g/mol	228.29	Formula: C_15_H_16_O_2_
Boiling point	°C	360.5	at 760 mmHg
Melting point	°C	158.5	at 760 mmHg
Density	g/cm^3^	1.195	at 25 °C
Relative density	--	1.2	at 25 °C
Flash point	°C	227	Closed cup
Auto-ignition temperature	°C	510	at 760 mmHg
Water solubility	mg/L	300	at 25 °C
Vapor pressure	mm Hg	4.0 × 10^−8^	at 25 °C
log Kow	-	3.32	
Henry’s Law Constant	atm-m^3^/mol	4.0 × 10^−11^ (est.)	at 25 °C
Dissociation constants (pka)	--	9.6, 11.3	at 25 °C

**Table 2:** Manufacturing plants of bisphenol A (BPA) in Taiwan.

Manufacturer	Production Location	Production Capacity (Kilo Tons/Year)	Comment
Nan Ya Plastic Co. (One company of Formosa Plastics Group)	Mailiao (Yunlin, Taiwan)	420 (460) ^1^	Since the late 1990s, there have been four production lines for BPA in central Taiwan, partly used to produce polycarbonate (PC) and epoxy in another company (Formosa Plastics Group).
Chang Chun Plastics Co. (One company of Chang Chun Group)	Daliao (Kaohsiung, Taiwan)	270	There are two production lines in southern Taiwan, which started to produce BPA in 2005 and 2009, respectively. Most of the BPA production is used to produce BPA-based products domestically.
Taiwan Prosperity Chem. Co.	Linyuan (Kaohsiung, Taiwan)	100 (107) ^1^	There has been one production line for producing BPA in southern Taiwan since 1995. This company was merged into Chang Chun Group in August 2021. Most of the BPA produced by the company was exported to the Asian countries.

^1^ The values in the parenthesis denote the production capacities after debottlenecking process.

**Table 3:** Statistics on the BPA production capacity per capita in 2019.

Region/Country	Production Capacity ^1^ (Tons)	Population ^2^ (Millions)	BPA Production per Capita (kg/Capita)
Belgium	225,000	11.5	19.565
Brazil	22,000	211.0	0.104
China	1,342,000	1433.8	0.936
Germany	351,000	83.5	4.204
Iran	26,000	82.9	0.314
Japan	364,000	126.9	2.868
The Netherlands	363,000	17.1	21.228
Poland	12,000	37.9	0.317
Russia	64,000	145.9	0.439
Saudi Arabia	215,000	34.3	6.268
Singapore	187,000	5.8	32.241
Republic of Korea	779,000	51.2	15,215
Spain	306,000	46.7	6.552
**Taiwan**	**653,000 (847,000) ^3^**	**23.8**	**27.437 (35.588)**
Thailand	342,000	69.6	4.914
USA	983,000	329.1	2.987

^1^ Source [2]. ^2^ Population in mid-2019 [40]. ^3^ Total production capacity after debottlenecking.

**Table 4:** Statistics on the values of import from and export in Taiwan for BPA since 2010 ^1^.

Year	Import (Metric Ton)	Export (Metric Ton)
2010	4289	392,385
2011	2348	320,694
2012	336	364,973
2013	1313	293,044
2014	7404	274,680
2015	1533	306,962
2016	710	237,384
2017	1510	193,236
2018	48	231,610
2019	1082	244,257
2020	414	268,320
2021	555	248,210
2022	2040	229,062

^1^ Source [30].

**Table 5:** Production amounts and release quantities of BPA in Taiwan ^1^.

Year	Production Amount (Metric Ton)	Release Amount (Metric Ton)		
		Discharge	Transfer	Total
2017	663,770.40	42.80	1.60	44.40
2018	712,571.40	79.35	0.30	79.65
2019	663,285.87	39.06	1.23	40.29
2020	663,285.87	55.93	0.56	56.49
2021	745,305.06	47.89	0.58	48.47

^1^ Source [31].

## Data Availability

The authors confirm that the data supporting the findings of this study are available within the article.
